# Supplementary material for: Design and Evaluation of an Outdoor Exercise Program for Pediatric Cancer Survivors
Source: Children (Basel). 2022 Jul 27;9(8):1117. doi: 10.3390/children9081117 (PMC9332767; doi:10.3390/children9081117)
Supplement: Supplementary file 1 [file children-09-01117-s001.zip › children-1813284-supplementary.pdf]

| EvaSys | Fragebogen Sportangebote [Copy] [Copy] [Copy] | Electric Paper |
|---|---|---|

Ruhr-Universität Bochum | ActiveOncoKids Zentrum Ruhr
Universitätsklinikum Essen | ActiveOncoKids Onlineumfrage

Bitte so markieren: ☐ ☒ ☐ ☐ ☐ Bitte verwenden Sie einen Kugelschreiber oder nicht zu starken Filzstift. Dieser Fragebogen wird maschinell erfasst.
Korrektur: ☐ ■ ☐ ☒ ☐ Bitte beachten Sie im Interesse einer optimalen Datenerfassung die links gegebenen Hinweise beim Ausfüllen.

Hallo liebe ActiveOncoKids Teilnehmer,

es freut uns, dass Du im Jahr 2019 an unseren Angeboten teilgenommen hast. Um uns im Jahr 2020 weiter zu verbessern und um Deine Wünsche zu berücksichtigen, bitten wir Dich, den folgenden Fragebogen auszufüllen. Dies nimmt ca. 10 Minuten in Anspruch.

Wenn Du mindestens 14 Jahre alt bist, kannst Du den Fragebogen alleine ausfüllen. Bist Du jünger als 14 Jahre, dann bearbeite den Fragebogen zusammen mit Deinen Eltern. Vielen Dank für die Unterstützung!

Hinweis: Aus Gründen der besseren Lesbarkeit wird auf die gleichzeitige Verwendung männlicher und weiblicher Sprachformen verzichtet. Sämtliche Personenbezeichnungen gelten für alle Geschlechter.

## 1.

1.1 Wer füllt den Fragebogen aus?
☐ Teilnehmer/in ☐ Teilnehmer/in zusammen mit den Eltern

## 2. Allgemeine Angaben

2.1 Wie alt bist Du?
☐ 7 Jahre oder jünger ☐ 8-12 Jahre ☐ 13-17 Jahre
☐ über 18 Jahre

2.2 Wie bist Du auf unser Angebot aufmerksam geworden?
☐ Broschüre ☐ Plakat ☐ Website
☐ E-Mail ☐ Mitarbeiter der ActiveOncoKids ☐ Arzt
☐ Ehemalige Teilnehmer ☐ Sportwissenschaftler der Kinderklinik ☐ Sonstiges

2.3 Sonstiges:

## 3. Aktivitäten

3.1 Du hast an einem/mehreren Schnuppertagen teilgenommen?
☐ Ja ☐ Nein

## 4. Schnuppertage

4.1 An welchen Schnuppertagen hast Du teilgenommen?
☐ Klettern ☐ Tauchen ☐ Segeln
☐ Windsurfen ☐ Stand-Up-Paddling & Kanu

## 5. Organisation

5.1 Waren die Informationen im Vorfeld der Schnuppertage ausreichend?
☐ Ja ☐ Nein

MUSTER



## 5. Organisation [Fortsetzung]

5.2 Nein, weil:

5.3 Wie bewertest du den Anmeldevorgang? sehr schlecht ☐ ☐ ☐ ☐ ☐ sehr gut

## 6. Ablauf des Schnuppertags

| | | stimme nicht zu | | | | stimme voll zu |
|---|---|---|---|---|---|---|
| 6.1 | Die Sportlehrer waren hilfsbereit | ☐ | ☐ | ☐ | ☐ | ☐ |
| 6.2 | Die Sportlehrer konnten die Sportart gut anleiten | ☐ | ☐ | ☐ | ☐ | ☐ |
| 6.3 | Die Sportlehrer haben meine Fragen ignoriert | ☐ | ☐ | ☐ | ☐ | ☐ |
| 6.4 | Die Inhalte wurden für mich verständlich vermittelt | ☐ | ☐ | ☐ | ☐ | ☐ |
| 6.5 | Ich habe mich schlecht betreut gefühlt | ☐ | ☐ | ☐ | ☐ | ☐ |
| 6.6 | Ich habe mich stets sicher gefühlt | ☐ | ☐ | ☐ | ☐ | ☐ |
| 6.7 | Ich habe mich schlecht beaufsichtigt gefühlt | ☐ | ☐ | ☐ | ☐ | ☐ |
| 6.8 | Auf meine Ängste wurde angemessen eingegangen | ☐ | ☐ | ☐ | ☐ | ☐ |
| 6.9 | Der Schnuppertag hat mir Spaß gemacht | ☐ | ☐ | ☐ | ☐ | ☐ |
| 6.10 | Ich konnte meine Wunschsportart(en) ausprobieren | ☐ | ☐ | ☐ | ☐ | ☐ |
| 6.11 | Es waren zu wenige Sportgeräte vorhanden | ☐ | ☐ | ☐ | ☐ | ☐ |
| 6.12 | Die Rahmenbedingungen waren gut (Umkleidemöglichkeiten, Aufenthaltsmöglichkeiten,...) | ☐ | ☐ | ☐ | ☐ | ☐ |

## 7. Dein persönliches Fazit

| | | stimme nicht zu | | | | stimme voll zu |
|---|---|---|---|---|---|---|
| 7.1 | Ich habe Fortschritte in den Sportarten gemacht | ☐ | ☐ | ☐ | ☐ | ☐ |
| 7.2 | Ich möchte die neu kennengelernte Sportart auch außerhalb der ActiveOncoKids betreiben | ☐ | ☐ | ☐ | ☐ | ☐ |
| 7.3 | Im Umfeld meines Wohnortes gibt es keine Möglichkeit die Sportart auszuüben | ☐ | ☐ | ☐ | ☐ | ☐ |
| 7.4 | Ich traue mir nun zu, die Sportarten auch ohne die Sportlehrer von ActiveOncoKids durchzuführen | ☐ | ☐ | ☐ | ☐ | ☐ |

7.5 Welche zusätzliche Unterstützung wünschst du dir, um die Sportart auch ohne die ActiveOncoKids durchzuführen?

## 8. Dein Befinden nach dem Schnuppertag

Am Ende des Schnuppertags fühlte ich mich:

| | | stimme nicht zu | | | | stimme voll zu |
|---|---|---|---|---|---|---|
| 8.1 | glücklich | ☐ | ☐ | ☐ | ☐ | ☐ |
| 8.2 | unangenehm erschöpft | ☐ | ☐ | ☐ | ☐ | ☐ |
| 8.3 | leistungsfähig | ☐ | ☐ | ☐ | ☐ | ☐ |
| 8.4 | lustlos | ☐ | ☐ | ☐ | ☐ | ☐ |
| 8.5 | zufrieden | ☐ | ☐ | ☐ | ☐ | ☐ |
| 8.6 | gestresst | ☐ | ☐ | ☐ | ☐ | ☐ |
| 8.7 | gut gelaunt | ☐ | ☐ | ☐ | ☐ | ☐ |
| 8.8 | selbstbewusst | ☐ | ☐ | ☐ | ☐ | ☐ |
| 8.9 | enttäuscht | ☐ | ☐ | ☐ | ☐ | ☐ |



MUSTER

MUSTER



## 8. Dein Befinden nach dem Schnuppertag [Fortsetzung]

| | | | | | | |
|---|---|---|---|---|---|---|
| 8.10 | motiviert Sport zu machen | ☐ | ☐ | ☐ | ☐ | ☐ |
| 8.11 | Ich hatte Schmerzen | ☐ | ☐ | ☐ | ☐ | ☐ |
| 8.12 | Ich war froh, dass es vorbei war | ☐ | ☐ | ☐ | ☐ | ☐ |
| 8.13 | Ich wäre gerne noch länger geblieben | ☐ | ☐ | ☐ | ☐ | ☐ |

## 9. Dein Fazit

9.1 Wie würdest du den/die Schnuppertag/e insgesamt bewerten? hat mir nicht gefallen ☐ ☐ ☐ ☐ ☐ hat mir sehr gut gefallen

## 10. Aktivitäten

10.1 Du hast an einer/mehreren Freizeiten teilgenommen? ☐ Ja ☐ Nein

## 11. Freizeiten

11.1 An welchen Freizeiten hast Du teilgenommen?

☐ Wassersportwochenende (Jugendliche und junge Erwachsene) ☐ Wassersportwochenende (Familienfahrt Münster) ☐ Tauchwochenende

## 12. Organisation

12.1 Waren die Informationen im Vorfeld der Freizeiten ausreichend? ☐ Ja ☐ Nein

12.2 Nein, weil:

12.3 Wie bewertest du den Anmeldevorgang? sehr schlecht ☐ ☐ ☐ ☐ ☐ sehr gut

## 13. Ablauf der Freizeit/en

| | | stimme nicht zu | | | | stimme voll zu |
|---|---|---|---|---|---|---|
| 13.1 | Die Sportlehrer waren hilfsbereit | ☐ | ☐ | ☐ | ☐ | ☐ |
| 13.2 | Die Sportlehrer konnten die Sportart gut anleiten | ☐ | ☐ | ☐ | ☐ | ☐ |
| 13.3 | Die Sportlehrer haben meine Fragen ignoriert | ☐ | ☐ | ☐ | ☐ | ☐ |
| 13.4 | Die Inhalte wurden für mich verständlich vermittelt | ☐ | ☐ | ☐ | ☐ | ☐ |
| 13.5 | Ich habe mich schlecht betreut gefühlt | ☐ | ☐ | ☐ | ☐ | ☐ |
| 13.6 | Ich habe mich stets sicher gefühlt | ☐ | ☐ | ☐ | ☐ | ☐ |
| 13.7 | Ich habe mich schlecht beaufsichtigt gefühlt | ☐ | ☐ | ☐ | ☐ | ☐ |
| 13.8 | Auf meine Ängste wurde angemessen eingegangen | ☐ | ☐ | ☐ | ☐ | ☐ |
| 13.9 | Die Freizeit hat mir Spaß gemacht | ☐ | ☐ | ☐ | ☐ | ☐ |
| 13.10 | Ich konnte meine Wunschsportart(en) ausprobieren | ☐ | ☐ | ☐ | ☐ | ☐ |
| 13.11 | Es waren zu wenige Sportgeräte vorhanden | ☐ | ☐ | ☐ | ☐ | ☐ |
| 13.12 | Die Rahmenbedingungen waren gut (Umkleidemöglichkeiten, Aufenthaltsmöglichkeiten,...) | ☐ | ☐ | ☐ | ☐ | ☐ |
| 13.13 | Bei der organisierten Anreise sind Probleme aufgetreten | ☐ | ☐ | ☐ | ☐ | ☐ |
| 13.14 | Die Unterkunft hat mir gut gefallen | ☐ | ☐ | ☐ | ☐ | ☐ |
| 13.15 | Ich habe mich in der Gruppe mit den anderen Teilnehmern wohl gefühlt | ☐ | ☐ | ☐ | ☐ | ☐ |
| 13.16 | Mit dem Essen war ich unzufrieden | ☐ | ☐ | ☐ | ☐ | ☐ |

## 14. Dein persönliches Fazit

| | | stimme nicht zu | | | | stimme voll zu |
|---|---|---|---|---|---|---|
| 14.1 | Ich habe Fortschritte in den Sportarten gemacht | ☐ | ☐ | ☐ | ☐ | ☐ |



MUSTER

MUSTER



## 14. Dein persönliches Fazit [Fortsetzung]

| | | | | | | |
|---|---|---|---|---|---|---|
| 14.2 | Ich möchte die neu kennengelernte Sportart auch gerne außerhalb der ActiveOncoKids betreiben | ☐ | ☐ | ☐ | ☐ | ☐ |
| 14.3 | Im Umfeld meines Wohnortes gibt es keine Möglichkeit die Sportart auszuüben | ☐ | ☐ | ☐ | ☐ | ☐ |
| 14.4 | Ich traue mir nun zu, die Sportarten auch ohne die Sportlehrer von ActiveOncoKids durchzuführen | ☐ | ☐ | ☐ | ☐ | ☐ |

14.5 Welche zusätzliche Unterstützung wünschst du dir, um die Sportart auch ohne die ActiveOncoKids durchzuführen?

## 15. Dein Befinden nach der Freizeit

Am Ende der Freizeit fühlte ich mich:

| | | stimme nicht zu | | | | stimme voll zu |
|---|---|---|---|---|---|---|
| 15.1 | glücklich | ☐ | ☐ | ☐ | ☐ | ☐ |
| 15.2 | unangenehm erschöpft | ☐ | ☐ | ☐ | ☐ | ☐ |
| 15.3 | leistungsfähig | ☐ | ☐ | ☐ | ☐ | ☐ |
| 15.4 | lustlos | ☐ | ☐ | ☐ | ☐ | ☐ |
| 15.5 | zufrieden | ☐ | ☐ | ☐ | ☐ | ☐ |
| 15.6 | gestresst | ☐ | ☐ | ☐ | ☐ | ☐ |
| 15.7 | gut gelaunt | ☐ | ☐ | ☐ | ☐ | ☐ |
| 15.8 | selbstbewusst | ☐ | ☐ | ☐ | ☐ | ☐ |
| 15.9 | enttäuscht | ☐ | ☐ | ☐ | ☐ | ☐ |
| 15.10 | motiviert Sport zu machen | ☐ | ☐ | ☐ | ☐ | ☐ |
| 15.11 | Ich hatte Schmerzen | ☐ | ☐ | ☐ | ☐ | ☐ |
| 15.12 | Ich war froh, dass es vorbei war | ☐ | ☐ | ☐ | ☐ | ☐ |
| 15.13 | Ich wäre gerne noch länger geblieben | ☐ | ☐ | ☐ | ☐ | ☐ |

## 16. Dein Fazit

16.1 Wie würdest du die Freizeit/en insgesamt bewerten? hat mir nicht gefallen ☐ ☐ ☐ ☐ ☐ hat mir super gefallen

## 17. Motivation für die Teilnahme

Ich habe beim Sportangebot der ActiveOncoKids Zentrum Ruhr teilgenommen, weil...

| | | stimme nicht zu | | | | stimme voll zu |
|---|---|---|---|---|---|---|
| 17.1 | Ich eine neue Sportart ausprobieren wollte | ☐ | ☐ | ☐ | ☐ | ☐ |
| 17.2 | Ich neue Leute kennenlernen wollte | ☐ | ☐ | ☐ | ☐ | ☐ |
| 17.3 | Ich mein Selbstbewusstsein stärken wollte | ☐ | ☐ | ☐ | ☐ | ☐ |
| 17.4 | Ich mein körperliches Wohlbefinden verbessern wollte | ☐ | ☐ | ☐ | ☐ | ☐ |
| 17.5 | Mein Arzt es mir empfohlen hat | ☐ | ☐ | ☐ | ☐ | ☐ |
| 17.6 | Meine Familie es mir empfohlen hat | ☐ | ☐ | ☐ | ☐ | ☐ |
| 17.7 | Ich kein anders Sportangebot kenne das zu mir passt | ☐ | ☐ | ☐ | ☐ | ☐ |
| 17.8 | Ich einen Buddy mitbringen durfte | ☐ | ☐ | ☐ | ☐ | ☐ |
| 17.9 | Ich Langeweile hatte | ☐ | ☐ | ☐ | ☐ | ☐ |
| 17.10 | Ich Probleme beim Sport habe | ☐ | ☐ | ☐ | ☐ | ☐ |
| 17.11 | Es kostenlos war | ☐ | ☐ | ☐ | ☐ | ☐ |

## 18. Barrieren beim Sporttreiben außerhalb der ActiveOncoKids

Wie sehr stimmst du folgenden Gründen zu, dass sie dich von der Teilnahme an sportlichen Aktivitäten im Verein oder in der Freizeit abhalten?

stimme nicht zu — stimme voll zu



MUSTER

MUSTER



## 18. Barrieren beim Sporttreiben außerhalb der ActiveOncoKids [Fortsetzung]

| | | | | | | |
|---|---|---|---|---|---|---|
| 18.1 | Ich habe keine Motivation im Allgemeinen | ☐ | ☐ | ☐ | ☐ | ☐ |
| 18.2 | Ich habe keine Motivation zu körperlich-sportlichen Aktivitäten bzw. Sport | ☐ | ☐ | ☐ | ☐ | ☐ |
| 18.3 | Ich sehe keinen Nutzen/Vorteil in der Teilnahme an sportlichen Aktivitäten | ☐ | ☐ | ☐ | ☐ | ☐ |
| 18.4 | Meine Familie unterstützt mich nicht bei der Teilnahme | ☐ | ☐ | ☐ | ☐ | ☐ |
| 18.5 | Meine Familie hat Bedenken bei der Teilnahme | ☐ | ☐ | ☐ | ☐ | ☐ |
| 18.6 | Die Belastung durch Schule/Ausbildung/Studium/Arbeit lässt keine Teilnahme zu | ☐ | ☐ | ☐ | ☐ | ☐ |
| 18.7 | Die Belastung durch die Therapie lässt keine Teilnahme zu | ☐ | ☐ | ☐ | ☐ | ☐ |
| 18.8 | Die zeitliche Belastung durch die Teilnahme ist zu hoch | ☐ | ☐ | ☐ | ☐ | ☐ |
| 18.9 | Die Informationen von Vereinen/Anbietern sind unzureichend | ☐ | ☐ | ☐ | ☐ | ☐ |
| 18.10 | Die Kosten sind zu hoch | ☐ | ☐ | ☐ | ☐ | ☐ |
| 18.11 | Ich habe generell wenig Interesse an Sport | ☐ | ☐ | ☐ | ☐ | ☐ |
| 18.12 | Meine körperliche Leistungsfähigkeit ist zu schlecht | ☐ | ☐ | ☐ | ☐ | ☐ |
| 18.13 | Ich habe Bedenken mich beim Sport zu verletzen | ☐ | ☐ | ☐ | ☐ | ☐ |
| 18.14 | Ich habe Bedenken nicht mit den anderen Mithalten zu können | ☐ | ☐ | ☐ | ☐ | ☐ |

## 19. Wünsche/Anmerkungen

19.1 Hast du Wünsche oder Anmerkungen zu den Angeboten der ActiveOncoKids Zentrum Ruhr (wie z.B. weitere Sportarten, Verbesserungsvorschläge,...)?

## 20. Ende

Vielen Dank für Deine Teilnahme!



MUSTER
